# Association of cardiotrophin-like cytokine factor 1 levels in peripheral blood mononuclear cells with bone mineral density and osteoporosis in postmenopausal women

**DOI:** 10.1186/s12891-020-03924-9

**Published:** 2021-01-11

**Authors:** Xuan Chen, Jianyang Li, Yunjin Ye, Jingwen Huang, Lihua Xie, Juan Chen, Shengqiang Li, Sainan Chen, Jirong Ge

**Affiliations:** 1 Key Research Laboratory of Osteoporosis Syndrome Genomics, Fujian Academy of Chinese Medical Sciences, No. 282 Wusi Road, Fuzhou, 350003 Fujian China; 2 College of Traditional Chinese Medicine, Fujian University of Traditional Chinese Medicine, Fuzhou, 350122 China

**Keywords:** Cardiotrophin like cytokine factor 1, Bone density, Osteoporosis, Fracture, Postmenopause

## Abstract

**Background:**

Recent research has suggested that cardiotrophin-like cytokine factor 1 (CLCF1) may be an important regulator of bone homeostasis. Furthermore, a whole gene chip analysis suggested that the expression levels of CLCF1 in the peripheral blood mononuclear cells (PBMCs) were downregulated in postmenopausal women with osteoporosis. This study aimed to assess whether the expression levels of CLCF1 in PBMCs can reflect the severity of bone mass loss and the related fracture risk.

**Methods:**

In all, 360 postmenopausal women, aged 50 to 80 years, were included in the study. A survey to evaluate the participants’ health status, measurement of bone mineral density (BMD), routine blood test, and CLCF1 expression level test were performed.

**Results:**

Based on the participants’ bone health, 27 (7.5%), 165 (45.83%), and 168 (46.67%) participants were divided into the normal, osteopenia, and osteoporosis groups, respectively. CLCF1 protein levels in the normal and osteopenia groups were higher than those in the osteoporosis group. While the *CLCF1* mRNA level was positively associated with the BMD of total femur (*r* = 0.169, *p* = 0.011) and lumbar spine (*r* = 0.176, *p* = 0.001), the protein level was positively associated with the BMD of the lumbar spine (*r* = 0.261, *p* < 0.001), femoral neck (*r* = 0.236, *p* = 0.001), greater trochanter (*r* = 0.228, *p* = 0.001), and Ward’s triangle (*r* = 0.149, *p* = 0.036). Both the mRNA and protein levels were negatively associated with osteoporosis development (*r* = − 0.085, *p* = 0.011 and *r* = − 0.173, *p* = 0.014, respectively). The association between CLCF1 protein level and fracture risk was not significant after adjusting for BMD.

**Conclusions:**

To our knowledge, this is the first clinical study to show that CLCF1 expression levels in the PBMCs of postmenopausal women can reflect the amount of bone mass or the severity of bone mass loss.

**Supplementary Information:**

The online version contains supplementary material available at 10.1186/s12891-020-03924-9.

## Background

Cardiotrophin-like cytokine factor 1 (CLCF1), also known as cardiotrophin-like cytokine (CLC), new neurotrophin-1 (NNT-1), or B-cell stimulatory factor-3 (BSF-3) [1], is a member of the IL-6 cytokine family. The gene encoding for CLCF1 is present on chromosome 11q13, and its sequence predicts a 225-aa protein with a 27-aa signal peptide, and a molecular mass of 22 kDa in the mature form [2]. CLCF1 requires the soluble cytokine receptor-like protein (CRLF1) or soluble tripartite ciliary neurotrophic factor receptor (sCNTFR) as a chaperone to be efficiently secreted. The dimer competes with ciliary neurotrophic factor (CNTF) or neuropoietin (NP) for binding to the CNTFR complex comprising CNTFR, glycoprotein (gp)130, and the leukemia inhibitory factor (LIF) receptor (LIFR) [3] and activates the Jak-STAT signaling cascade.

CLCF1 is broadly expressed in major cell types and organs and has diverse functions. It was first identified in lymph nodes and the spleen and found to induce body weight loss and B-cell hyperplasia along with increases in serum IgG and IgM levels [2]. Recently, an important role for CLCF1 in hematopoiesis regulation was further established with a bias toward myeloid cell differentiation [4]. CLCF1 is also known as a potent neurotrophic factor and a neuroendocrine modulator of pituitary corticotroph function. It is expressed in fetal neuroepithelial cells and induces astrocyte differentiation [5] and is required for motoneuron survival during development [6]. Neonate mice with null mutations of *Clcf1* were reported to have died from motor neuron loss affecting the facial nucleus and ventral horn of the lumbar spinal cord [7]. *CLCF1* mutation in humans leads to a cold-induced sweating syndrome 2, which is characterized by profuse sweating after exposure to cold as well as congenital physical abnormalities of the head and spine. In addition, CLCF1 was identified as an injurious factor in the human renal disease focal segmental glomerulosclerosis [8].

Although several functions of CLCF1 have been recognized in different tissues under physiologic and pathologic conditions, many questions persist about its role in vivo. CLCF1 and its chaperone CRLF1 were detected in the developing murine skeleton and cultured murine primary osteoblasts [9, 10]. However, the function of CLCF1 in bone homeostasis is barely known. In this study, we sought to investigate the involvement of CLCF1 in this context, based on a previous work in which we demonstrated that *CLCF1* could be a responding gene correlated with therapeutic effects on postmenopausal osteoporosis [11]. In a whole gene chip analysis of peripheral blood mononuclear cells (PBMCs) of women with postmenopausal osteoporosis, we found modulation of transcripts as well as changes in the expression of a set of genes not previously correlated with bone metabolism, of which the *CLCF1* gene was the most downregulated [12].

Based on the previous evidence, some issues need to be elucidated. Whether the expression level of the *CLCF1* gene in PBMCs can reflect the amount of bone mass or the severity of bone mass loss in postmenopausal women remains to be clarified along with whether it can reflect the associated fracture risk. Hence, this study aimed to evaluate the relationship between the expression level of the *CLCF1* gene in PBMCs and bone mass or fracture risk in 360 elderly female participants who had been through menopause for more than 2 years.

## Methods

### Participants and data collection

The data for this study was obtained from the specialist osteoporosis clinic of Fujian Academy of Chinese Medical Sciences; 392 postmenopausal women who visited the hospital from January 2012 to December 2018 were enrolled in this study. The inclusion criteria were as follows: 1) patients aged 50 to 80 years; 2) patients who had been through menopause for more than 2 years; 3) patients living in Fuzhou City for more than 10 years; and 4) patients who signed the informed consent form. The following patients were excluded: 1) patients with secondary osteoporosis or diseases that affect bone metabolism; 2) patients who had used traditional Chinese medicine to treat osteoporosis in the month before study recruitment, hormone replacement therapy or calcitonin in the 3 months before study recruitment, or bisphosphonates and other drugs for 15 consecutive days in the 6 months before study recruitment.

A survey to assess the patients’ health status, measurement of bone mineral density (BMD), routine blood test, and CLCF1 expression level test were conducted. The questionnaire and experimental data were computerized by a researcher who did not participate in the questionnaire survey, according to double-blind entry rules, and then verified by a designated person. Questionnaires in which more than 5% of the data were missing were excluded. Eventually, 360 participants were found eligible for inclusion in this study.

### Health examination survey

The questionnaire adopted was designed by the research group for this study and is included in the additional file. It included questions on age, age of menarche, age of menopause, frequency of pregnancy, number of children breastfed, gastrointestinal diseases (no, yes), hypertension (no, yes), coronary artery disease (CAD; no, yes), awareness of osteoporosis (no, yes) and calcium supplementation (no, yes), history of fracture (no, yes), and body mass index calculation based on height and weight. The survey was administered by professionally trained personnel via a face-to-face interview with the participants.

### BMD measurement

BMD of the orthotopic lumbar vertebrae (L2~4) and left hip was measured using the Discovery W dual-energy X-ray bone densitometer (Hologic Corporation, USA; coefficient of variation: 1.0 CV%, accuracy: 0.25%). Bone health was determined using the T-score criteria of the World Health Organization: normal, T-score ≥ − 1; osteopenia, − 2.5 < T-score < − 1; and osteoporosis, T-score ≤ − 2.5 [13].

### Blood sample collection

Blood samples were collected in the morning after fasting for at least 8 h. The routine blood test was performed immediately after the samples were collected, and PBMCs were separated using lymphocyte separation medium (human) (Cat. P8610, Solarbio, China) and stored at − 80 °C for real-time quantitative PCR and western blot analysis.

### Real-time reverse transcription PCR

The total RNA in PBMCs was extracted with the Trizol one-step method (Cat. 10296010, Invitrogen, USA). Then, the purity and concentration of the RNA solution were determined using a spectrophotometer (NanoDrop 2000, Thermo Fisher Scientific, USA), and the integrity was determined by 1% formaldehyde denatured agarose gel electrophoresis. One microgram of the total RNA was taken from each sample for reverse transcription by using RevertAid First Strand cDNA Synthesis Kit (Cat. K1622, Fermentas, USA), and the resulting cDNA was stored at − 80 °C. Each sample was quantitatively analyzed using SYBR© Premix Ex TaqTM GC Kit (Cat. No.RR430, Takara, Japan) with ABI Prism Fast 7500 system (Applied Biosystems, USA). Cycling conditions were as follows: initiation at 95 °C for 30 s, turned 40 cycles at 95 °C for 5 s, and switching to 60 °C for 30 s thereafter. Using GAPDH as an internal control, each sample was analyzed in triplicate and the average was obtained. The relative quantitative method of the double standard curve was used for the quantitative analysis of genes. The relative mRNA expression level of the target gene of each sample was calculated using the following formula: relative mRNA expression = target gene expression / internal control expression. The primers were synthesized by Takara Biotech and are listed below:

- *CLCF1-*forward: 5′-TTG GAG GTG CCC TAT AAA CCA GAA -3′.
- *CLCF1-*reverse: 5′-GTT TGC CAC TCT GTG CTT TGG A -3′.
- *GAPDH-*forward: 5′-GGG AAA CTG TGG CGT GAT -3′.
- *GAPDH-*reverse: 5′-GAG TGG GTG TCG CTG TTG A − 3′.

### Western blot analysis

The appropriate amount of protein lysate and 1% volume of protease inhibitor were added in the tubes with PBMCs on the ice to obtain total protein. After measuring the protein concentration, 50 μg of total protein of each sample was obtained and separated by 10% polyacrylamide gel electrophoresis. The separated proteins were transferred to a polyvinylidene difluoride membrane and then sealed with 50 g/L skim milk for 2 h. A diluted primary antibody (ab251886, Abcam, USA) and the corresponding diluted secondary antibody (Beyotime Institute of Biotechnology, China) were added in turn to probe the CLCF1 protein and the internal control, GAPDH. Finally, a hypersensitive ECL kit (Cat. 35,055, Thermo Scientific, USA) was used to detect the results with a gel imaging system (FluorChem M, ProteinSimple, USA), and the band was statistically analyzed.

### Statistical analysis

The continuous variables that follow a normal distribution are expressed as mean ± standard deviation (SD) values. Two-sample independent *t*-test was performed to for the compare two groups, and one-way ANOVA was used to compare three groups. If there were statistical differences among the groups, the least significant difference (LSD) method was used for post hoc analysis. The CLCF1 mRNA and protein data did not follow a normal distribution, while the CLCF1 protein data followed a normal distribution after log-transformation. Continuous variables that do not follow a normal distribution are expressed as median (interquartile range, IQR) values. The comparison between two groups was performed using the Mann-Whitney *U* test, and the comparison among the three groups was performed using the Kruskal-Wallis H test followed by LSD post hoc tests for pairwise comparisons. The categorical variables are expressed as frequency (%), and the comparisons between groups were performed using Chi-square or Fisher’s exact test when appropriate. If the differences between the groups were statistically significant, Bonferroni post hoc tests were used for pairwise comparisons. Binary univariate logistic regression (Enter method) was used to analyze the relationship between CLCF1 and fractures, hypertension, and coronary heart disease, and variables with significant differences in the univariable analyses were incorporated into a multiple logistic regression analysis (forward-LR method). Pearson or Spearman correlation analysis was used to determine the relationship between CLCF1 and BMD, osteoporosis, and gastrointestinal diseases. Statistical analyses were performed using SPSS 21.0 (IBM, Armonk, NY, USA). All statistical tests were two-sided, and the significance threshold of *p*-value was set to 0.05.

## Results

### Baseline characteristics of the participants

Table 1 describes the basic characteristics of the participants, including demographics, menstruation, reproduction, BMD, and routine blood test findings. A total of 360 participants were included in this study. Based on bone health, the participants were divided into three groups: 27 (7.5%) in the normal group, 165 (45.83%) in the osteopenia group, and 168 (46.67%) in the osteoporosis group. The age of the osteoporosis group was higher than that of the osteopenia group. Menopausal age differed among the groups, but the difference between any two groups was not significant. The distribution of gastrointestinal diseases was also different among the three groups. The frequencies of gastrointestinal diseases in the osteopenia group and the osteoporosis group were higher than that in the normal group, while the frequency in the osteoporosis group was higher than that in the osteopenia group. The distribution of hypertension among the three groups was different. The frequency of hypertension in the normal group was higher than that in the osteopenia group and the osteoporosis group.

**Table 1:** Baseline characteristics of participants^a^

	Normal group	Osteopenia group	Osteoporosis group	*p* Value
Number	27	165	168	–
Age (year)	62 (59,66)	61 (57,65)	63 (59,66.75) ^c^	0.002
ATA (year)	15 (13,16)	15 (14,16)	15 (14,17)	0.364
Frequency of pregnancy	2 (1,3)	3 (2,3)	2 (1,4)	0.128
Number of children breastfed	1 (1,2)	1 (1,2)	1 (1,2)	0.222
ATM (year)	52 (49,54)	50 (49,53)	50 (47,52)	0.019
BMI (kg/m^2^)	24.26±2.66	23.74±3.11	23.26±2.83	0.146
Milk-drinking habits	19 (70.4%)	127 (77%)	122 (72.6%)	0.582
Calcium supplementation	13 (48.1%)	88 (53.3%)	76 (45.2%)	0.334
Gastrointestinal diseases	4 (14.8%)	50 (30.3%) ^b^	69 (41.1%) ^b, c^	0.010
Hypertension	10 (37%)	25 (15.2%) ^b^	22 (13.1%) ^b^	0.006
CAD	1 (3.7%)	5 (3.0%)	5 (3.0%)	0.979
WBC (10^9 /L)	5.51 (4.58,6.23)	5.4 (4.5,6.22)	5.495 (4.5,6.2)	0.904
TLC (10^9 /L)	2 (1.63,2.41)	1.9 (1.53,2.31)	1.9 (1.6,2.2)	0.602
RBC (10^12 /L)	4.5 (4.18,4.63)	4.35 (4.01,4.62)	4.4 (4.11,4.59)	0.412
HGB (g/L)	134.23±8.35	133.43±10.59	136.76±9.37	0.053
PLT (10^9 /L)	213 (198.5242)	224 (186.25,263)	223 (188.75,254)	0.901

^a^Values are presented as n (%), mean ± SD or median (interquartile range, IQR), *ATA* age at menarche, *ATM* age at menopause, *BMI* body mass index, *CAD* coronary artery disease, *WBC* white blood cell count, *TLC* total lymphocyte count, *RBC* red blood cell count, *HGB* hemoglobin, *PLT* blood platelet count. ^b^ *p* < 0.05 compared with the normal group. ^c^ *p* < 0.05 compared with the osteopenia group

### Relationship between bone health and CLCF1 expression

**Fig. 1:**
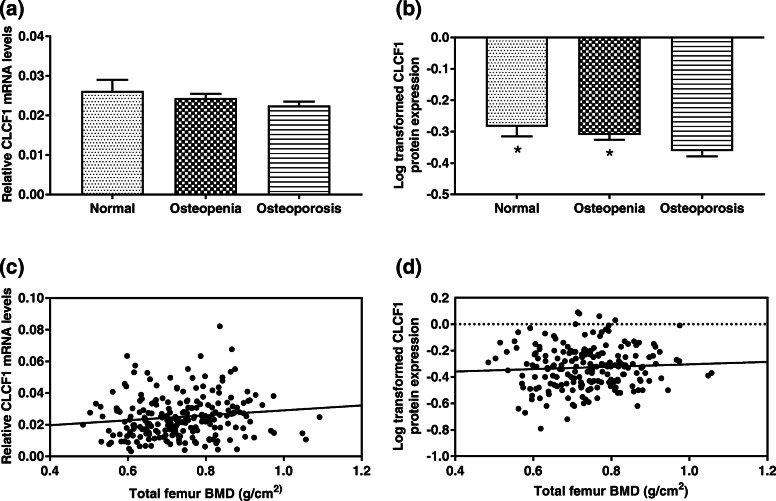
Relationship between bone health and (**a**) *CLCF1* mRNA levels; (**b**) log-transformed CLCF1 protein (^−^x ± SD). *p < 0.05 compared with the osteoporosis group using one-way ANOVA and LSD post-hoc test. Relationship between total femur BMD and (**c**) *CLCF1* mRNA levels; (**d**) log-transformed CLCF1 protein (^−^x ± SD)

The Kruskal-Wallis H test was used to compare the differences in *CLCF1* mRNA levels in different groups, and there was no significant difference among the groups (Fig. 1a). One-way ANOVA with the LSD post-hoc test was performed to compare the difference in log-transformed CLCF1 protein expression between groups, and the results suggested that the log-transformed CLCF1 protein expression levels in the normal group and the osteopenia group were both higher than that in the osteoporosis group (Fig. 1b).

Spearman’s rank correlation analysis was performed to calculate the correlation between *CLCF1* mRNA levels and the BMD of the total femur and showed that *r* = 0.169, *p* = 0.011, suggesting that CLCF1 mRNA levels were positively associated with BMD (Fig. 1c). Pearson’s correlation analysis was used to calculate the correlation between log-transformed CLCF1 protein level and the BMD of the total femur and showed that *r* = 0.061, *p* = 0.395, suggesting that log-transformed CLCF1 protein expression was positively associated with the BMD of the total femur, but the difference was not statistically significant (Fig. 1d).

### Relationship between CLCF1 expression levels and BMD at different sites and the occurrence of osteoporosis

We further analyzed the relationship between CLCF1 expression levels in PBMCs and BMD at different sites. Spearman’s rank correlation analysis was performed, and the results suggested that the *CLCF1* mRNA levels were positively associated with the BMD of the lumbar spine and negatively associated with the BMD of the femoral neck, greater trochanter, and Ward’s triangle (Table 2). Pearson’s correlation analysis was performed, and the results suggested that log-transformed CLCF1 protein expression was positively associated with the BMD of the lumbar spine, femoral neck, greater trochanter, and Ward’s triangle (Table 2).

**Table 2:** Results of correlation analysis between CLCF1 expression levels, and BMD levels in different parts^a^

Variables		Log-transformed CLCF1 protein	CLCF1 mRNA levels
Lumbar spine BMD	r	0.261	0.176
	*p*	<0.001	0.001
Femoral neck BMD	r	0.236	-0.141
	*p*	0.001	0.008
Greater trochanter BMD	r	0.228	-0.163
	*p*	0.001	0.002
Ward’s triangle BMD	r	0.149	-0.195
	*p*	0.036	<0.001
Bone health^b^	r	-0.173	-0.085
	*p*	0.014	0.011

^a^*BMD* bone mineral density; ^b^Since the normal, osteopenia, and osteoporosis groups were assigned values of 0, 1, and 2, respectively, a lower bone health number corresponds to better bone health

The normal, osteopenia, and osteoporosis groups were assigned values of 0, 1, and 2, respectively, and the relationship between the CLCF1 expression levels and the occurrence of osteoporosis was analyzed using Pearson’s correlation analysis. The results suggested that the CLCF1 mRNA levels and log-transformed protein expression were negatively associated with the occurrence of osteoporosis (Table 2); thus, the lower the CLCF1 mRNA and protein expression levels, the higher the possibility of osteoporosis.

### Relationship between CLCF1 expression levels and fracture occurrence

**Fig. 2:**
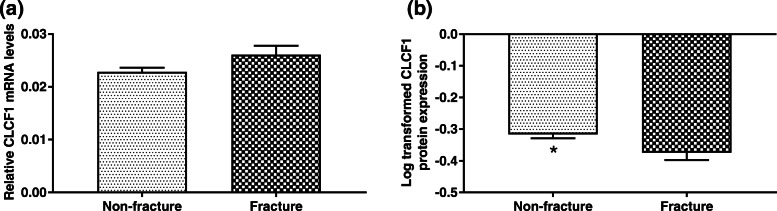
Relationship between bone fracture and (**a**) *CLCF1* mRNA levels; (**b**) log-transformed CLCF1 protein (^−^*x* ± SD). *p < 0.05 compared with the lowest category using two independent samples *t* test

The Mann-Whitney *U* test was performed to compare the *CLCF1* mRNA levels between the non-fracture and fracture groups, and there was no significant difference in *CLCF1* mRNA levels between the two groups (Fig. 2a). An independent-sample *t*-test was performed to compare the log-transformed CLCF1 protein expression levels between the two groups, and the log-transformed CLCF1 protein expression level in the non-fracture group was higher than that in the fracture group (Fig. 2b).

We set the fracture as the dependent variable and related factors as covariables, and then performed univariate logistic regression analysis (Enter method). The result suggested that CLCF1 protein level was protective factor for fractures (Table 3), i.e., the higher the expression levels, the less likely fractures will occur. However, when variables with significant differences in the univariable analyses were incorporated into multiple logistic regression analysis (forward-LR method), the expression level of CLCF1 protein did not have a direct relationship with the occurrence of fractures after adjusting for the influence of confounding factors. Instead, CLCF1 served as a protective factor in the occurrence of fractures through BMD, especially femoral neck BMD.

**Table 3:** Results of binary logistic regression analysis of fracture-related factors^a^

Variables	Univariate logistic regression			Multiple logistic regression		
	OR	(95% CI)	*p*	OR	(95% CI)	*p*
Constant	–	–	–	8.923	–	0.051
Lumbar spine BMD	0.197	(0.021, 1.876)	0.158			
Femoral neck BMD	0.101	(0.010, 0.979)	0.048	0.004	(0.000, 0.114)	0.001
Greater trochanter BMD	0.017	(0.001, 0.230)	0.002			
Ward’s triangle BMD	0.116	(0.015, 0.909)	0.040			
Total femur BMD	0.045	(0.002, 1.177)	0.063			
CLCF1 mRNA	5,111,738.706	(0.030, 8.7E+ 14)	0.110			
CLCF1 protein	0.096	(0.010, 0.908)	0.041			

^a^*OR* odds ratio, *CI* confidence interval of OR, *BMD* bone mineral density

### Influence of other diseases on the CLCF1 expression level

Due to differences in the distribution of diseases such as gastrointestinal diseases and hypertension among different groups, we further analyzed the influence of gastrointestinal diseases, hypertension, and coronary heart disease on the expression levels of CLCF1 mRNA and protein in PBMCs. Spearman’s rank correlation analysis was performed. The results suggested that CLCF1 mRNA and log-transformed protein expression levels had no significant correlation with gastrointestinal diseases (*r* = − 0.075, *p* = 0.287 and *r* = 0.004, *p* = 0.936 respectively).

The occurrence of hypertension was significantly correlated with age, and the ages of participants in different groups showed certain differences. Therefore, hypertension was set as a dependent variable, while age, *CLCF1* mRNA, and CLCF1 protein levels were set as covariables, after which a multiple logistic regression analysis (Enter method) was performed. The results suggested that age was a risk factor for hypertension (*p* < 0.05), while the mRNA levels and protein expression of CLCF1 were not correlated with hypertension (*p* > 0.05) (Supplementary Table 1).

CAD was set as a dependent variable, while age, CLCF1 protein and mRNA levels, and hypertension were set as covariables, and a multiple logistic regression analysis (Enter method) was performed. The results suggested that age was an independent risk factor for CAD (*p* < 0.05), and the mRNA levels and protein expression of CLCF1 were not correlated with CAD (*p* > 0.05) (Supplementary Table 1).

## Discussion

The skeletal system is one of the most important systems in the human body and bone homeostasis is maintained through bone remodeling in adults [14]. Osteoporosis is a major disease that is believed to be the result of a disparity between resorption and formation rates during remodeling. It is characterized by a progressive decline in BMD, which results in a dramatic increase in fracture risk for elderly individuals, especially in postmenopausal women. Some evidence suggests that morphological and functional changes in PBMCs, most of which are lymphocytes and monocytes, are closely related to the bone metabolism and may reflect the severity of osteoporosis in postmenopausal women [15].

Several studies have demonstrated that CLCF1 is involved in bone development and metabolism [1, 3]. The expression level of CLCF1 in PBMCs was observed to decrease in women with postmenopausal osteoporosis in a whole gene chip analysis [11]. Therefore, participants were collected in this study to investigate whether the expression level of CLCF1 in PBMCs can reflect the changes of BMD. Although the expression level of *CLCF1* mRNA in PBMCs showed a lower trend in the group with lower bone mass, the difference was not statistically significant. The CLCF1 protein level in the osteoporosis group was significantly lower than that in the normal and osteopenia groups. Our analysis of BMD at different sites in 206 local postmenopausal women showed that, femoral BMD could better reflect the decline of bone health than lumbar spine BMD, with the increase of age [16]. To avoid the false negative diagnosis in late old women caused by ectopic calcification, hyperosteogeny and vertebral compression in lumbar spine, the relation between the total femur BMD and CLCF1 expression was analyzed. Correlation analysis showed that the expression level of *CLCF1* mRNA was positively correlated with the total femur BMD. Analysis of CLCF1 expression levels and BMD in different parts of the body also suggested that the *CLCF1* mRNA level was positively correlated with lumbar spine BMD, and the CLCF1 protein level was positively correlated with the BMD of the lumbar spine, femoral neck, greater trochanter, and Ward’s triangle. Moreover, the lower the expression levels of CLCF1 mRNA and protein, the higher the possibility of osteoporosis.

Furthermore, the osteoporosis-related decline in bone mass and changes in bone architecture and mechanical properties led to decreased physical activity and frailty with an increased risk of fractures [17]. Therefore, the relationship between the expression level of CLCF1 and the fractures risk was further analyzed. The results showed that after excluding the influence of bone density, a decrease in CLCF1 expression level did not directly lead to an increase in fracture incidence. Considering the differences in age and the incidences of gastrointestinal diseases and hypertension among different groups, we analyzed the effects of gastrointestinal disease, hypertension, and CAD on the expression level of CLCF1 in PBMCs. The results showed that the expression level was independent of the above factors.

To our knowledge, this is the first study to elucidate the positive correlation between the *CLCF1* gene expression level in PBMCs and BMD and the negative correlation between it and osteoporosis occurrence. Members of the IL-6 cytokine family play prominent roles in health and disease and can influence various tissue metabolic processes, where they often act as diagnostic or prognostic indicators of disease activity and response to therapy [18]. Our study provides clinical evidence demonstrating that the *CLCF1* gene expression level in PBMCs may reflect BMD in elderly women, especially the role of the CLCF1 protein level in indicating BMD of the lumbar spine, femoral neck, greater trochanter, and Ward’s triangle, all of which are the most likely skeletal sites of osteoporosis and fracture [17].

However, there are several important limitations of the current study that must be noted. First, because this was an observational study and we only measured CLCF1 levels at one time point, we cannot determine the true causal relationship between CLCF1 expression levels and BMD. Because this study included only postmenopausal women in Fujian Province of China and the bone mass loss of postmenopausal women in different regions and ethnic groups varied greatly, these findings may not be generalizable. Furthermore, we cannot exclude any unmeasured bias or confounding factors, and as such, these findings should be interpreted cautiously until they are replicated in other studies. Finally, the role of the *CLCF1* gene in bone metabolism remains elusive although the results suggest that higher expression levels of the *CLCF1* gene in PBMCs are accompanied by higher BMD and a lower incidence of osteoporosis.

Some cytokines of the IL-6 family, such as cardiotrophin 1 (CT-1), oncostatin M (OSM), and LIF, have been reported to play critical roles in bone cell biology as stimuli for both bone formation pathways acting through the LIFR and gp130 [19]. Like other members of the IL-6 family, it is generally understood that the CLCF1 compound cytokines act by similar mechanisms, involving the use of a complex including LIFR, gp130, and an α receptor subunit, in this case, CNTFR [1]. Formation of this signaling complex results in JAK phosphorylation followed by phosphorylation of STAT proteins, especially STAT3, and activation of SHP2/Ras/MAPK signaling. The positive correlation between the CLCF1 expression level and BMD in our study suggests that the gene may play a stimulating role in bone remodeling just like some of the family members. Both CLCF1 and *CRLF1* mRNA levels in osteoblasts are stimulated by parathyroid hormone [9], a hormone that stimulates bone formation depending on gp130 signaling within the osteoblast lineage. Deletion of either gp130 or LIFR results in a neonatal lethal skeletal phenotype of low bone mass, high osteoclast number, and low osteoblast number [20, 21]. Mice with osteoblast-specific disruption of the *Stat3* gene showed an osteoporotic phenotype because of a reduced bone formation rate [22]. All these results for the upstream or downstream signal molecules of CLCF1 also provide some evidence for our hypothesis. Surprisingly, however, slight inhibition of mineralization in primary calvarial osteoblasts was observed after treatment with 10 ng/mL CLCF1 for 4 days [10]. The results of another study indicate that CLCF1 binds mouse mesenchymal stem cells, triggers STAT1 and 3 phosphorylation, inhibits the upregulation of master genes involved in the control of osteogenesis, and markedly prevents osteoblast generation and mineralization [3]. Since both of the conclusions contradicting our hypothesis are based solely on the data of cell experiments in vitro, it remains unclear whether the same conclusions will be reached in vivo, which involves a much more complex environment and more interactions than in vitro conditions.

## Conclusions

In summary, our study provides the preliminary clinical evidence demonstrating that decreased CLCF1 expression levels in PBMCs of postmenopausal women are closely associated with decreased BMD and osteoporosis occurrence, especially that of the lumbar spine, femoral neck, greater trochanter, and Ward’s triangle. However, there is still much to be learned about the roles of the CLCF1 cytokine in bone function and metabolism, and further studies are needed to obtain this information.

## Supplementary Information

**Additional file 1: Supplementary Table 1.** Results of multiple logistic regression analysis of the correlation of CLCF1 expression levels with hypertension and CAD.

**Additional file 2: Supplementary Dataset 1.** Questionnaires data of the participants and results of the BMD measurement, routine blood test, and CLCF1 expression level test.

**Additional file 3.** Osteoporosis investigation and research questionnaire (The fifth edition). The questionnaire used in our study was developed for this study by our research group.

## Data Availability

All data generated or analyzed during this study are included in this published article and its supplementary information file (Additional file 2.xlsx Supplementary Dataset 1).

## Abbreviations

BMD: Bone mineral density
BSF-3: B-cell stimulatory factor-3
CAD: Coronary artery disease
CLC: Cardiotrophin-like cytokine
CLCF1: Cardiotrophin-like cytokine factor 1
CNTF: Ciliary neurotrophic factor
CRLF1: Cytokine receptor-like protein
CT-1: Cardiotrophin 1
LIFR: Leukemia inhibitory factor receptor
NNT-1: New neurotrophin-1
NP: Neuropoietin
OSM: Oncostatin M
PBMCs: Peripheral blood mononuclear cells
sCNTFR: soluble tripartite ciliary neurotrophic factor receptor
