# Supplementary material for: Association of cardiotrophin-like cytokine factor 1 levels in peripheral blood mononuclear cells with bone mineral density and osteoporosis in postmenopausal women
Source: BMC Musculoskelet Disord. 2021 Jan 11;22:62. doi: 10.1186/s12891-020-03924-9 (PMC7798196; doi:10.1186/s12891-020-03924-9)
Supplement: Supplementary file 3 — Additional file 3. Osteoporosis investigation and research questionnaire (The fifth edition). The questionnaire used in our study was developed for this study by our research group. [file 12891_2020_3924_MOESM3_ESM.pdf]

Osteoporosis Investigation and Research Questionnaire (The fifth edition)

Random number: _____

Blood sample number: _____

Participant: _____

Researcher: _____

Record date: _____

First visit □ Follow-up visit □

Identification number □□□□□□□□□□□□□□□□□□

Home address: ____

Home phone: ____

Mobile phone number: ____

Birthplace: ____

Date of birth: ____

Occupation: Worker□ Farmer□ Cadre□ Intellectual□ Unemployed□

Nature of the work: Physical labor □ Mental labor □

Education: None□ Primary school□ Middle school□ High school□ Technical secondary school□ Junior□ College□ Graduate□

Gender: Female□ Male□

Living Habits:

Cigarette smoking: Daily□ Has quit □ Never□

Tea drinking: Daily□ Occasionally□ Never□

Coffee drinking: Daily□ Occasionally□ Never□

Milk drinking: Daily□ Occasionally□ Never□

Sun exposure time: ≥3h□ 3~1h□ <1h□

Type of exercise: Tai Chi□ Walking□ Setting-up exercise□ Running□ Dancing□ Ball games□

Time for exercise: ≥3h□ 3~1h□ <1h□

Exercise frequency: ≥5 times a week□ 3~1 times a week□ <3 times a week□

Years of participating in sports: _____

Exercise intensity: High intensity□ Moderate intensity□ Low intensity□

Medical History

Gastrointestinal disease: Yes (Gastritis□ Gastric ulcer□ Duodenal Ulcer□ Other: _____) Unknown□ No□

Hypertension: Yes□ Unknown□ No□

Coronary artery disease: Yes□ Unknown□ No□

Mellitus: Insulin dependent diabetes mellitus□ Non-insulin-dependent diabetes mellitus□ Unknown□ No□

Other disease: Yes□ (_________) Unknown□ No□

The history of bone fracture after menopause: Yes□ (Age: _____, Fracture site: ____) No□

Knee Osteoarthritis: Yes□ Unknown□ No□

Calculus: Yes□ (Site: ________) Unknown□ No□

Marital and Reproductive History

Marital status: Married□ Unmarried□

Age at menarche: ____

Time of pregnancy: ____

Time of abortion: ____

Number of children breastfed: ____

Other Information

Age at menopause: ____

Current height: ____m

Height when young: ____m

Body weight: ____kg

BMI: ____kg/m^2

Blood pressure: ____/____ (mmHg)

Awareness of OP: Yes□ No□

Recent treatment of osteoporosis: Yes□ No□

Calcium supplementation: Yes□ (____mg) No□
